# Role of Inflammation and the NF-κB Signaling Pathway in Hirschsprung’s Disease

**DOI:** 10.3390/biom14080992

**Published:** 2024-08-12

**Authors:** Enas Zoheer Elkrewi, Ahmad A. Al Abdulqader, Rasul Khasanov, Silke Maas-Omlor, Michael Boettcher, Lucas M. Wessel, Karl-Herbert Schäfer, María Ángeles Tapia-Laliena

**Affiliations:** 1 Department of Pediatric Surgery, Medical Faculty of Mannheim, University of Heidelberg, Theodor-Kutzer-Ufer 1–3, 68167 Mannheim, Germany; 2 Department of Surgery, College of Medicine, King Faisal University, Al Hofuf 31982, Saudi Arabia; 3 Working Group Enteric Nervous Systems (AGENS), University of Applied Sciences Kaiserslautern, Amerikastrasse 1,66482 Zweibrücken, Germanykarlherbert.schaefer@hs-kl.de (K.-H.S.)

**Keywords:** Hirschsprung’s disease (HSCR), Enteric Nervous System (ENS), NF-κB pathway, inflammation, neuronal migration

## Abstract

Hirschsprung’s disease (HSCR, incidence 1/5000 live births) is caused by the failure of neural crest-derived precursors to migrate, survive, proliferate, or differentiate during the embryonic development of the Enteric Nervous System (ENS), which could be disrupted by many factors, including inflammatory processes. The NF-κB family controls several biological processes, including inflammation, neurogenesis, and cell migration. With the aim of studying the potential role of NF-κB in HSCR, we have analyzed the expression of the NF-κB main subunits and other NF-κB-related genes by RT-qPCR in HSCR tissue samples (sub-divided into ganglionic and aganglionic segments). We found decreased gene expression of the NF-κB main subunit *RELA* but also of *NFKBIA*, *TNFA*, *TFGBR2*, and *ERBB3* in the pathologic distal aganglionic segments compared to the proximal ganglionic segments. Moreover, we could also confirm the lower protein expression of RelA/p65 in the aganglionic distal segments by immunofluorescence staining. Further, we show that the expression of RelA/p65 protein in the proximal segments concurs with lymphocyte infiltration in the bowel tissue, indicating a pro-inflammatory activation of p65 in the proximal ganglionic HSCR tissue in the patients analyzed. All in all, our findings suggest that the modulation of NF-κB signaling in the neuro-enteric system does obviously contribute to the pathological effects of HSCR.

## 1. Introduction

Hirschsprung’s disease (HSCR, incidence 1/5000) or congenital megacolon is characterized by a local or general reduction or complete absence of the intrinsic gastrointestinal innervation, with individual variations from a local aganglionosis of the most distal colonic segments to a total aganglionosis [1,2]. Here, the Enteric Nervous System (ENS) is completely absent or at least severely affected, resulting in varying grades of a- or hypoganglionosis. The compromised ENS leads to the impossibility of the intrinsic muscles to relax, while extrinsic innervation is still intact, thus resulting in a distal stenosis that impairs defecation and might lead to fatal co-morbidities such as toxic megacolon and enterocolitis.

HSCR is caused by a colonization failure of enteric precursor cells derived from the neural crest (EPCs) to proliferate, migrate, survive, or differentiate during ENS formation [1,3]. The regulation of this process is critical, and many different genes and proteins are involved in both migratory and colonization processes [3]. Regarding genetics, HSCR shows a 4:1 male predominance and a clear increased HSCR risk with Down syndrome [1,4]. The first HSCR-linked gene was *RET* kinase [5], followed by endothelin receptor B (*EDNRB*) [6]. Furthermore, the combination of both mutations was reported to cause highly penetrant distal aganglionosis [7]. However, in a prenatal diagnosis study [8], fetuses carrying a *RET* variant did not develop any HSCR symptoms after years of follow-up. Thus, though the *RET* gene is autosomal dominant, its mutation shows incomplete penetrance and does not always lead to Hirschsprung’s diagnosis [8]. In addition, a wide spectrum of mutations affecting many different genes (*Plesin, ErbB, NTKR3, L-1CAM*, etc.) has been associated with HSCR, confirming the multigenic inheritance and partial penetrance of the syndrome [9,10,11,12]. Nevertheless, the occurrence and severity of HSCR in many cases still remain unexplained by the genetics [11,12]. Thus, Hirschsprung is a multifactorial disease, although many genes influence HSCR occurrence, environmental factors could also impact the risk [1,9].

The ENS is closely linked to the local immune system, gastrointestinal macrophages, and dendritic cells within the intestinal wall. Neuroimmunological interactions and communications may be responsible for modulating physiological functions of the gastrointestinal tract (GIT), such as motility [13]. The local immune system is in turn influenced by the microbiome [14] and also influences the plasticity of the ENS [15].

It is known that the ENS is not only affected by GIT disorders; it can be equally affected by systemic diseases such as diabetes, cancer, or neurodegenerative diseases [16,17]. For example, patients suffering from Parkinson’s disease (PD) are known to be affected by motility disorders or gastric emptying disorders as the disease progresses [18]. A hypothesis that PD has its first site of manifestation in the GIT is becoming increasingly established [19]. The brain and intestine are closely connected via the so-called brain–gut axis, and processes that take place in the intestine can also influence the brain [19,20,21].

The NF-κB pathway consists of a family of transcription factors that can be found in most cells of the central and peripheral nervous systems, mainly as NF-κB1/p50 homodimers and NF-κB1/RelA heterodimers [22], which function as transcriptional activators in the canonical pro-inflammatory pathway [23,24,25].

Indeed, the NF-κB pathway plays an important role in the structural and functional development of the nervous system [22,26]. Embryonic neurogenesis, neural progenitor migration and differentiation, as well as synaptic signaling, neuroprotection, and neural plasticity, are particularly regulated by the NF-κB system [27,28,29].

Inflammatory and immune responses through NF-κB signaling are known to be implicated in many nervous system illnesses, including neurodegenerative disorders such as Parkinson’s, Alzheimer’s, and Huntington’s diseases, multiple sclerosis, and neurodevelopmental diseases such as Hirschsprung’s disease [16,30]. Consequently, NF-κB signaling has been proposed as a therapeutic target for inflammatory neurodegeneration [31,32,33].

In addition, the inflammatory environment-specific immune cells (macrophages, dendritic cells) are a significant source of pro-inflammatory cytokines, including IFNγ, IL-1, and TNFα, which induce inflammation through the NF-κB pathway [34,35]. Moreover, the inflammation itself plays a role in neurostimulation and enteric neuronal migration [36,37,38,39,40], as well as in neuroregeneration through the NF-κB pathway [41].

Altogether, the NF-κB pathway appears to be a relevant pathway for enteric neuronal migration and survival, which suggests that it may also be important in HSCR disease development. Therefore, in this study, we have analyzed the expression of the main subunits of the NF-κB pathway, *RELA* and *NF-KB1*, together with other NF-κB-related and pro-inflammatory factors, on HSCR patient’s samples with the aim of analyzing their potential role in Hirschsprung’s disease.

## 2. Materials and Methods

### 2.1. Ethical Approval and Samples Collection

The collection and use of patient material have been performed according to informed consent signed by patients’ parents and approved by the “Medizinische Ethik-Kommission II” of the Medical Faculty Mannheim, University of Heidelberg (2011-237N-MA). Samples have only been identified by sequential code numbers with no other identifying details.

Colon tissue segments from Hirschsprung’s patients (27 samples) and from non-HSCR surgeries (8 samples from anastomosis, included as internal controls to validate the assays in other intestinal tissue) were obtained from the Pediatric Surgery Clinic at the University Hospital Clinic Mannheim, Germany.

Samples were divided into segments (A, B, C, D, etc.) indicating progressive HSCR pathology, from the proximal end (closer to the stomach, a ganglionic healthy segment with normal innervation) to the distal end (closer to the rectum, an aganglionic segment with pathological innervation). Each division was cut again in two parts, where one piece was immediately frozen in iso-pentane at (−80 °C) and then used for RT-qPCR analysis, and the other piece was shortly washed in PBS and fixed in 4% paraformaldehyde (PFA) for 24 h, followed by paraffin embedment for immunohistochemical staining.

For the further experiments, in each HSCR patient, the A segments (closer to the stomach) were considered “proximal, ganglionic” and compared to their corresponding distal segment (closer to the rectum, usually D or afterwards), which were labeled “distal, aganglionic with pathological innervation”. 

Regarding the non-HSCR tissue used as experimental controls, each sample remained as a whole and was not subdivided. From the 8 non-HSCR samples collected, 7 were eligible for RT-PCR and only 5 had the quality required for IHC staining.

### 2.2. RT-qPCR

27 HSCR patients were analyzed, where the distal segments (closer to the rectum, an aganglionic segment with pathological innervation) were compared to the proximal segments (closer to the stomach, a ganglionic healthy segment with normal innervation). Furthermore, we also included 7 non-HSCR samples (from anastomosis surgeries) as internal calibrator for the calculations. The list of primers used in the assay is summarized in Table 1.

Tissue samples were diced using a TissueLyser (Qiagen, Valencia, CA, USA), and total RNA was extracted using TRIsure™ (BIO-38032, Bioline, Meridian Biosciences, OH, USA) and Rneasy Micro Kit (74004, Qiagen, Valencia, CA, USA) following the manufacturer´s instructions. The RNA concentration was measured in the Infinite M200 microplate reader (Tecan Group Ltd., Männedorf, Switzerland).

cDNA conversion was processed using a BioScript™ Reverse Transcriptase kit (BIO-27036, Bioline, Meridian Biosciences, OH, USA) and Random hexamer primers (BIO-38028, Bioline, Meridian Biosciences, OH, USA). The synthesis was carried out using a peqSTAR Thermocycler (PeqLab Biotechnology GmbH, Erlangen, Germany) as follows: 5 min denaturation at 70 °C, 10 min annealing at 20 °C, 60 min elongation at 40 °C, and 10 min inactivation at 70 °C.

RT-qPCR reactions were performed with the SensiFAST^TM^ SYBR Lo-ROX Kit from (BIO-94020, Bioline, Meridian Biosciences, OH, USA) using the QuantStudio 5 device (Applied Biosystems Inc., Foster City, CA, USA) as follows: 2 min at 50 °C, initial denaturation 10 min at 95 °C; 40 × cycles of (denaturation 15 s at 95 °C, annealing 1 min at 55 °C), followed by a final Melting Curve of 15 s at 95 °C, 1 min at 55 °C, 15 s at 95 °C.

All experiments were performed in triplicate. The comparative 2^−ΔΔCt^ method was used to calculate gene expression, where data were first normalized to *GAPDH* as the houskeeping standard (dCt: Target Ct—Housekeeping Ct). Then, for each gene sample ddCt (ddCt: Sample dCt—Calibrator dCt) was calculated using the average of the 7 non-HSCR controls as a calibrator. Finally, fold 2^−ΔΔCt^ was calculated for each gene. An example of amplification plot can be found in Figure S1.

### 2.3. Immunohistochemistry

Colon samples of 25 HSCR patients (subdivided into proximal and distal segments; total: 50 samples) were analyzed.

Moreover, because the expression levels of p65 and p50 in our cohort of HSCR patients were unknown, 5 tissue samples from non-HSCR surgeries (anastomosis) were included in the study to validate the antibodies in non-HSCR intestinal tissue.

Tissue sections from proximal (A) and distal (from D on) segments were cut at 3 μm thickness using a microtome (RM2245, Leica Microsystems GmbH, Wetzlar, Germany).

Briefly, samples were de-paraffinized and re-hydrated through serial washes in xylene (5 min × 2 times), ethanol (100% 2 min × 2, 90% 2 min, 80% 2 min, 70% 1 min), and PBS (3 min) (Sigma-Aldrich, St. Louis, MO, USA). After the HIER (heat antigen epitope retrieval) of 30 min in sodium citrate buffer (pH 6.0) (CL009C-100, DCS Innovative Diagnostik-Systeme, Hamburg, Germany), samples were permeabilized using 0.5% Triton X100-PBS (Sigma-Aldrich, St. Louis, MO, USA) for 10 min, then washed in PBS for 5 min, and blocked in 10% normal goat serum (NGS, X0907, AgilentDako, Santa Clara, CA, USA) in PBS at room temperature (RT) for 1 h. Next, sections were incubated for 1 h at RT with the corresponding anti-NF-κB subunit antibody along with Tubulin Beta III.

After 3 × 5 min washes in PBS 0.005%Tween 20 (Sigma-Aldrich, St. Louis, MO, USA), the secondary fluorescence antibody was added for 1 h at RT. Nuclear staining was conducted using DAPI (9542, Sigma-Aldrich, St. Louis, MO, USA) in PBS, 1:1000 for 3 min, followed by 3× washes in PBS for 5 min each. Finally, samples were briefly rinsed in distilled water, and directly mounted on Dako Fluorescence Mounting Medium (S3023; Agilent Dako, Santa Clara, CA, USA). All samples were stored at 4 °C in the darkness until image acquisition.

The following antibodies (all in concentration 1:500) were used: NF-κB/p65 (sc-8008, Santa Cruz Biotechnology Inc., Santa Cruz, CA, USA), NF-κB1 p105/50 (D4P4D) (#13586, Cell Signaling Technology, Danvers MA, USA), Anti-Beta III Tubulin Antibody Alexa Fluor^®^ 488 Conjugate (AB15708A4, Millipore Sigma, St. Louis, MO, USA), Anti-Tubulin β3 isoform Antibody (MAB1637, Millipore Sigma, St. Louis, MO, USA), Alexa Fluor^®^ 488 (Goat Anti-Mouse #A-10667, Molecular Probes, Invitrogen, Life Technologies Corp., Carlsbad, CA, USA), Alexa Fluor^®^ 568 (Goat Anti-Rabbit #A-11011, Goat Anti-Mouse #A-11004, Molecular Probes, Invitrogen, Life Technologies Corp., Carlsbad, CA, USA).

In parallel, tissue sections of 5 HSCR patients were co-stained using NF-kB/p65 (sc-8008, Santa Cruz Biotechnology Inc., Santa Cruz, CA, USA) and the Leukocyte Common Antigen/CD45 (GA751, Agilent Dako, Santa Clara, CA, USA) following the protocol described above. Double-staining reagent (LD591R015 AP polymer, anti-mouse; DCS Innovative Diagnostik-Systeme, Hamburg, Germany) was used for 30 min at RT. Then, instead of the secondary fluorescence antibody, samples were incubated with Fast Red Bright Red (HK182-5KE, Biogenex Laboratories, Fremont, CA, USA) for 20 min at 37 °C and finally mounted on Aquerous Mounting Medium (EL017R 120, Agilent Dako, Santa Clara, CA, USA).

### 2.4. Image Acquisition and Analysis

Pictures of the fluorescence-stained samples were taken using a confocal laser scanning microscope (TCS SP8, Leica Microsystems GmbH, Wetzlar, Germany) at 40× magnification.

Samples stained with fast red reagent were pictured using an inverted phase-contrast microscope (BZ-9000, KEYENCE, Corporation of America, Itasca, IL, USA) at objective magnifications of ×20 and ×60.

The image quantification was performed using Image J (version IJ 1.53a, National Institutes of Health, Bethesda, MD, USA). The integrated density was calculated for p65 (red) and Tubulin Beta III (green) in both the proximal and distal sections of each sample. At least 3–6 pictures (×40) per HSCR section were quantified in each sample. In total, 300 images were analyzed for each antibody staining (p65 and Tubulin Beta III).

### 2.5. Statistical Analysis

Mann–Whitney U test (Wilcoxon rank-sum test) was used to determine whether there was a statistically significant difference in the gene and protein expression levels between HSCR proximal (ganglionic) and distal (aganglionic) tissue samples. The F.N. Test was used to compare differences in gene expression between distal and proximal segments of HSCR patients. Differences were considered statistically significant at *p*-value ≤ 0.05.

## 3. Results

### 3.1. HSCR Proximal Ganglionic Segments Show Increased RELA and Pro-Inflammatory Gene Expression Profiles

**Figure 1:**
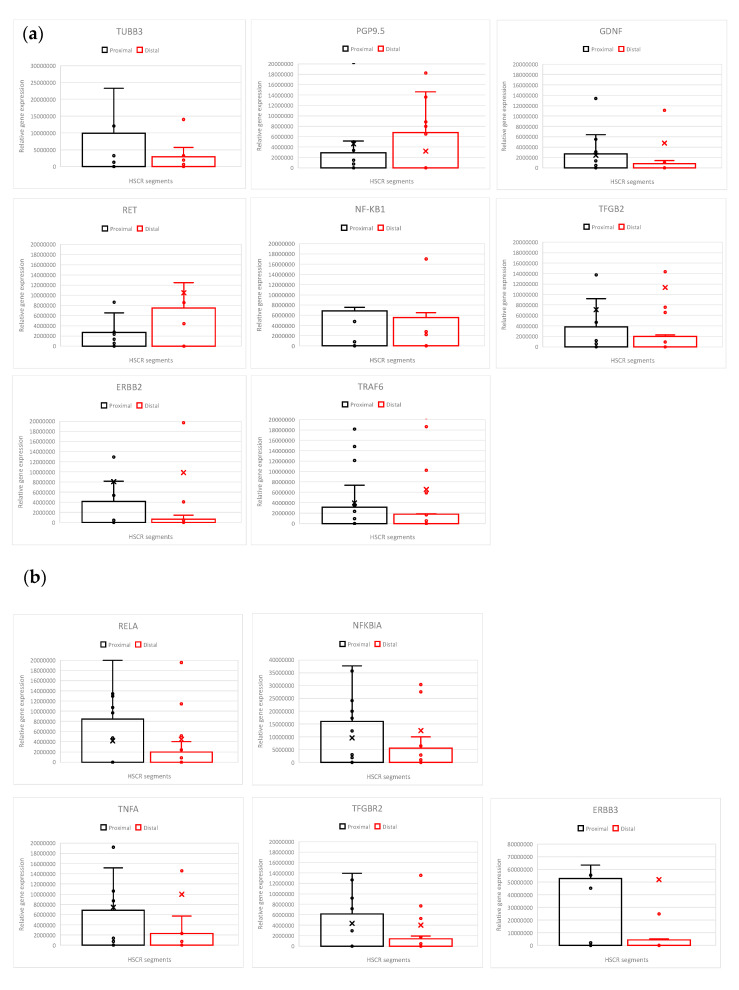

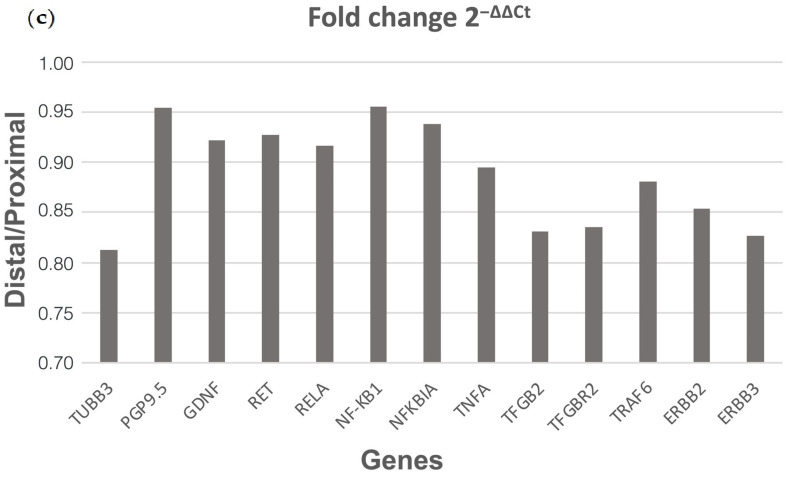
Comparison of gene expression by RT-qPCR analysis in the HSCR samples (distal segments relative to the proximal segments). All values were normalized to the average Ct values of the internal reference gene *GAPDH*. (**a**) Expression of genes encoding the neuronal markers (*TUBB3* and *PGP9.5*), genes related to neuronal development (*RET* and *GDNF*), and genes encoding *TGFB2, TGFBR2, ERBB2*, and *ERBB3*. (**b**) Expression of genes encoding NF-κB subunits *RELA* and *NF-KB1*, and NF-κB-related genes, *NFKBIA*: Inhibitor of NF-κB (F.N. Test *p* = 0.0004), *TNFA* (F.N Test *p* = 0.0006), and *TRAF6*. (**c**) Fold change comparison of 2^−ΔΔCt^ from distal to proximal samples (Distal/Proximal).

Firstly, with the aim of searching for the differences between the most pathologic intestinal HSCR distal sections (called aganglionic due to the uncompleted innervation, segments closer to the rectum) and the healthier HSCR proximal sections (ganglionic, segments closer to the stomach), we have examined the expression of genes that participate in or are related to the NF-κB pathway, together with neuronal markers (Figure 1a,b) in a cohort of 27 HSCR patients by RT-qPCR.

The analyzed genes were NF-κB pathway genes (*RELA, NF-κB1*, *NFKBIA* (NF-κB Inhibitor Alpha, IκBα), Tumor Necrosis Factor-Alpha (*TNFA*), and TNF Receptor-Associated Factor 6 (*TRAF6*), and other NF-κB pathway-related genes (the transforming growth factor Beta 2 (*TGFB2*), the transforming growth factor Beta 2 receptor (*TGFBR2*), *ERBB2* (HER-2/neu) and *ERBB3*), and also neuronal and glia markers together with HSCR-associated genes (*TUBB3*, *PGP9.5, RET, GDNF*) [53].

Briefly, our results show a decrease in *RELA* gene expression in the distal (aganglionic) segments of HSCR patients compared to the proximal (ganglionic) ones. In addition, the expression of the NF-κB inhibitor (*NFKBIA*), the pro-inflammatory cytokine (*TNFA*), and TFGBR2 was slightly decreased in the distal segment (Figure 1b).

We also observed lower *ERBB3* levels in the distal segments compared to the proximal ones (Figure 1b).

Though about 50% of HSCR patients do not express the *RET* gene [5], we did not detect *RET* loss in the HSCR cohort analyzed in our study (Figure 1a).

Furthermore, no significant alterations were obtained in the expression of the other analyzed genes (*GDNF, TRAF6, NF-κB1, ERBB2*, and *TGFB2*) between the proximal and distal HSCR segments (Figure 1b).

If we compare the fold 2^−ΔΔCt^ change in distal samples with respect to the proximal ones (Figure 1c), there is also a decrease in most of the pro-inflammatory gene expression (*RELA, TNFA, TGFB2, TRAF6*) and *ERBB3* in the distal sections (Figure 1c).

Altogether, we observed that the proximal ganglionic HSCR segments present higher levels of *RELA* and other pro-inflammatory factors than the distal aganglionic sections, which, maybe as a secondary effect, also present fewer or dysfunctional enteric neurons.

### 3.2. RelA/p65 Protein Levels Are Higher in HSCR Proximal Ganglionic Segments

**Figure 2:**
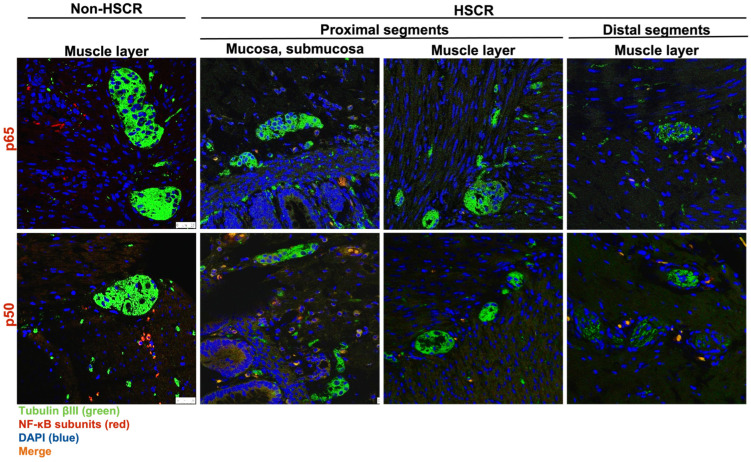
Immunohistochemistry of RelA/p65 and NFκB1/p50 in the proximal (mucosa, submucosa, and muscle layer) and distal (muscle layer) colon segments of a Hirschsprung patient. RelA/p65 and NFκB1/p50 were stained in red, neurons were labeled with TubβIII in green. A non-HSCR sample (muscle layer) was included as staining control. White bars: scale bar (25 μm).

To corroborate the RT-qPCR results, we examined the protein expression of the main subunits involved in the NF-κB canonical activation pathway, RelA/p65 and NF-κB1/p50. Here, 25 of the previous HSCR patients (again subdivided into proximal and distal segments) were analyzed by immunohistochemistry staining, where TubulinβIII was used as an internal control for neuronal markers (Figure 2). In addition, we also stained further 5 non-Hischprung intestinal sections as internal assay controls.

**Figure 3:**
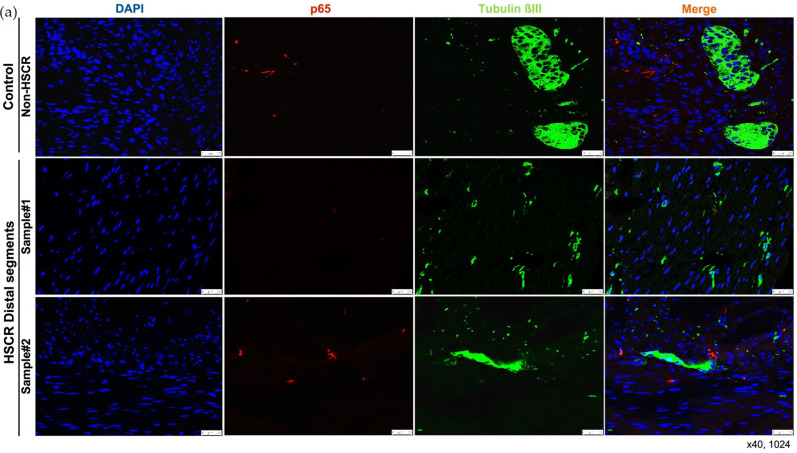

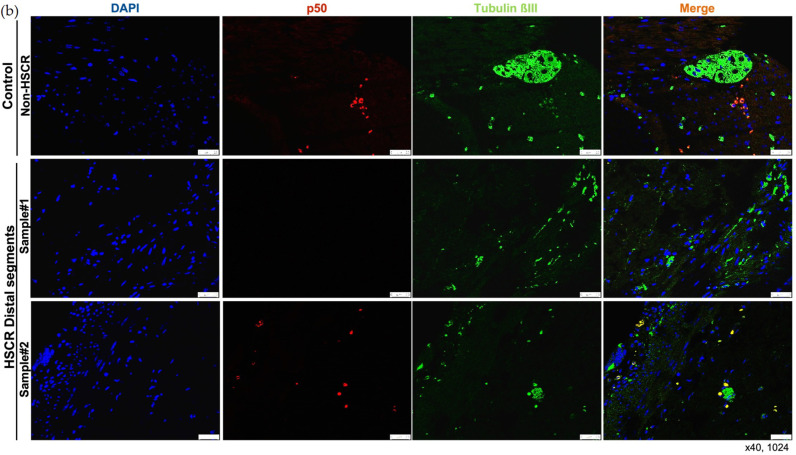
Immunohistochemistry of RelA/p65 (**a**) and NFκB1/p50 (**b**) in the muscle layers of the proximal and the distal colon segments of a Hirschsprung patient compared to a non-HSCR tissue. RelA/p65 and NFκB1/p50 were stained in red, neurons were stained with TubβIII in green. Images obtained with confocal laser scanning microscope (Leica TCS SP8), objective magnification ×40 (25 μm), resolution (XY): 1024 × 1024. White bars: scale bar (25 μm).

**Figure 4:**
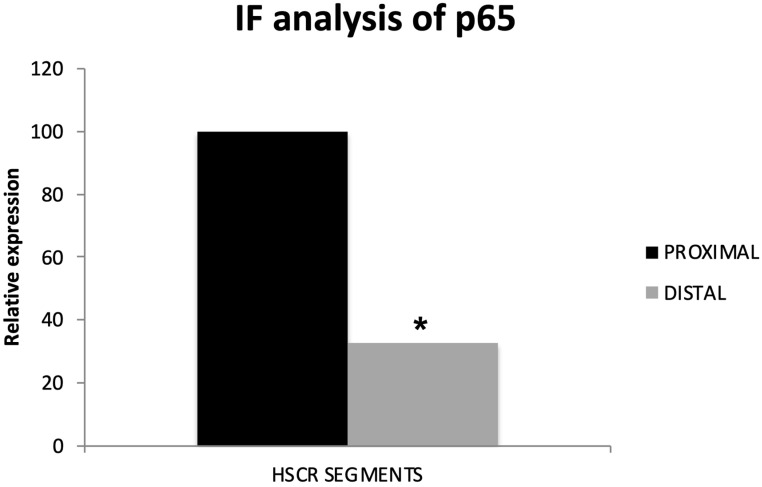
Quantification of RelA/p65 protein expression in distal samples HSCR compared to proximal HSCR samples. * Statistical significance cut-off (*p* < 0.003).

While we did not detect any remarkable difference on the levels of NF-κB1/p50 between the distal and proximal segments, the expression of the main NF-κB subunit, RelA/p65, was lower in the distal segments compared to the proximal HSCR colon tissue samples (Figure 3 and Figure 4), which confirms our previous RT-qPCR results.

Despite the expression of Tubulin βIII (Figure 2 and Figure 3), a neuronal marker, was not completely lost in the distal segments of all the HSCR patients, we could histologically observe that the innervations stained by Tubulin βIII were either not complete or were not forming healthy ganglia, indicating a non-complete enteric innervation in those patients.

Regarding the muscle layer, we observed variable immuno-reactivities of RelA/p65 (Figure 3a) and NF-κB1/p50 (Figure 3b), which indicates a highly heterogeneous expression of NF-κB proteins among HSCR patients. However, the NF-κB expression within the muscle layer of most of the HSCR samples was low or undetectable. In general, both RelA/p65 and NFκB1/p50 were mainly detected in the mucosa and submucosa layers of the colon wall (Figure 3).

Searching for accurate results, we quantified the immunofluorescence intensity from the pictures taken of the distal and proximal colon segments of HSCR patients. Again, we could prove that proximal HSCR samples present higher amounts of RelA/p65 protein than distal HSCR sections (*p* < 0.05) (Figure 4).

### 3.3. RelA/p65 Expression in HSCR Tissue Correlates with Lymphocyte Infiltration

Since RelA/p65 was mostly detected in the mucosal and submucosal layers, we wanted to confirm if this expression was related to tissue-infiltrating lymphocytes. Thus, we compared RelA/p65 protein expression with the expression of the leukocyte common antigen (LCA) in the distal segments of selected HSCR patients.

**Figure 5:**
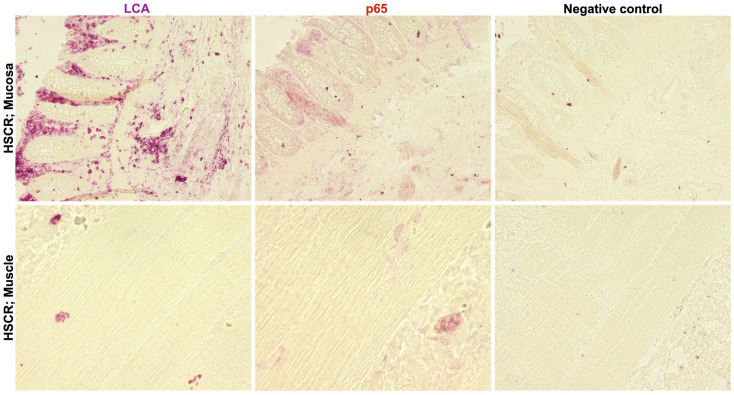
Immunohistochemistry of RelA/p65 (red) and LCA (leukocyte common antigen) (purple) in the distal (mucosa and muscle layer) colon segments (mucosa and muscle layer) of a Hirschsprung patient. RelA/p65, Mouse mAb (1:500) and LCA, Mouse mAbs (1:100) in fast red. Images obtained with Inverted fluorescence microscope (KEYENCE, Corporation of America, Itasca, IL, USA), Objective magnification ×20 (50 μm) and ×60 (12.5 μm). Images were taken from the same area of tissue. The “Negative control” was only stained with fast red with no primary antibody.

Results showed a co-expression of both proteins in the tissue, indicating a high expression of RelA/p65 in the tissue-infiltrated lymphocytes in the submucosa and mucosa layers. Again, no RelA/p65 expression was observed in the muscle layer (Figure 5).

Hence, proximal ganglionic HSCR segments present an inflammatory status, as suggested by RelA/p65 and LCA levels.

## 4. Discussion

These observations indicate a higher expression of *RELA* and other pro-inflammatory factors (*TNFA, TFGBR2*) in the HSCR proximal sections but a lower inflammation in the HSCR distal segments, which contain either less quantity or dysfunctional enteric neurons. The slightly higher expression of both the NF-κB inhibitor IκBα (*NFKBIA*) and the higher *RELA* concur in the proximal segments. Though it seems paradoxical, the regulation of the pathway is complex, with several interacting inhibitory IκB subunits and further 5 NF-κB subunits in constant exchange [23,24].

Additionally, we found lower *ERBB3* levels in the distal segments. Supporting this, *ERBB3* was previously reported to be deregulated in enteric neuropathies [54].

Also, we detected a co-expression of RelA/p65 and LCA, particularly in the mucosa and not in the muscle layer of HSCR proximal samples, indicating lymphocyte infiltration. Here, it could also be that the infiltration rate is correlated to dilatation and thus a result of a defect in the mucosal barrier.

Thus, our observed RelA/p65 levels and pro-inflammatory status of the HSCR proximal segments may contribute to the neurodegeneration that leads to the neuronal loss and impairment observed in the HSCR distal segments.

Consistently with our results, previous studies have already pointed out the relation of the NF-κB pathway to enteric neuronal survival. For instance, in a mouse model of HSCR (a model with a mutation in c-Ret, the major susceptibility gene in Hirschsprung’s disease), the impaired phosphorylation of NF-κB was pointed to as the possible cause of neurodegeneration of the spiral ganglion neurons (SGNs) in the inner ears and subsequent syndromic deafness [55]. In another mouse model of Parkinson´s Disease (PD), a debilitating neurodegenerative disorder, NF-κB inhibition prevented the loss of enteric neurons induced by inflammation [56].

Concerning the infiltration of pro-inflammatory macrophages, it has been associated with myenteric neuron injury, while their depletion helped to rescue the enteric neurons [57,58]. Moreover, impaired lymphocyte function has been associated with Hirschsprung-related enterocolitis [59,60]. In addition, post-surgical dysfunction of intestinal smooth muscle and enteric neurons has been attributed to inflammation and increased expression of TNF-α, IL-6, and IL-1α [61]. Importantly, NF-κB has been implicated in enteric neuronal loss by mediating 5-Fluorouracil intestinal inflammation and activating enteric glial cells [62], which also supports our hypothesis of a pro-inflammatory neurodegenerative role of NF-κB in HSCR development.

Considering the high availability of specific NF-κB inhibitors [25,63] and pro- and anti-inflammatory drugs on the market, new treatments based on the NF-κB pathway seem promising in the short term for the prevention and therapy of HSCR. Thanks to the critical contribution of NF-κB signaling to other severe diseases and cancer progression [23], the pharmaceutical industry has worked on many NF-κB inhibitors, with many already on the market or undergoing clinical trials [64]. However, in some cases, their way to the market was restricted due to toxic side effects (i.e., IKK inhibitors), indicating that systemic blockade of NF-κB may not be well tolerated [64,65]. Lately, subunit-specific inhibitors (i.e., anti-c-Rel, IT-901) showed better results and toleration in animal models [65]. Another simpler alternative to complete NF-κB inhibition could be targeting specific essential upstream activators/downstream effectors of the pathway, such as mTOR or AKT [65]. Other possibilities are drugs not directly related to the NF-κB pathway, but that anyway block it as a secondary effect. As an example, glatiramer acetate [66] or Evolocumab, a PCSK9 inhibitor) [67], have both been reported to reduce neuroinflammation by inhibiting NF-κB activation.

Natural compounds may also solve this problem. There is a large list of plant extracts, mostly flavonoids and polyphenols, that are known to inhibit NF-κB and ameliorate the inflammation, even also in neuronal dis eases, such us: ginger [68] and polygalacic acid in the treatment of Alzheimer’s disease (AD) [69]; or orientin, a phenolic phytoconstituent, in PD [70]. Curcumin is another well-known NF-κB modulator that can also revert neuroinflammation [71,72]. Of special interest here, for the treatment of a gastrointestinal disease like HSCR, is the flavonoid balicalein, which not only decreases inflammation but also regulates the microbiota [73].

Our observations set the stage for further studies on the role of NF-κB in neuro-enteric development. Modulation of NF-κB can be integrated with neural stem cell and regenerative research to potentiate neural progenitor migration and differentiation and optimize the outcomes of stem cell transplantation. Furthermore, NF-κB manipulation in animal models of HSCR as well as healthy animals, to confirm in vivo its effect on the enteric nervous system, would allow better disease characterization.

Given the intricate etiology of HSCR disease, the complex interaction between genetic and environmental factors, the varying severity, and the lack of treatment, currently the only available solution is surgery, which, indeed, comes with its complications. More extensive investigation of the NF-κB pathway is warranted to elucidate the molecular mechanisms underlying the pathogenesis of the disease.

Finally, screening programs for the use of anti-inflammatory drugs in pregnant women would provide useful data that can be utilized in correlative studies with the incidence of HSCR and other neurodevelopmental disorders. They also help in directing the discovery of potential disease mediators and biomarkers, not only for diagnosis and treatment, but also for strategies for disease prevention.

## 5. Conclusions

Altogether, the NF-κB pathway and inflammation seem to play an important role in the fate of the enteric nervous system and, therefore, in the development of Hirschsprung’s disease. Further studies are needed to determine if any of the current NF-κB modulators under clinical trials may be the new hope for Hirschsprung’s patients.

## Figures and Tables

**Table 1:** List of primers.

Gene Name	Forward Primers Sequence 5′ → 3′	Reverse Primers Sequence 5′ → 3′	References
*hGAPDH*	GCACCGTCAAGGCTGAGAAC	TGGTGAAGACGCCAGTGGA	[42]
*hTUBB3*	GCCTCTTCTCACAAGTACGTG	CCCCACTCTGACCAAAGATGAA	[43]
*hPGP9.5*	AAGGCCAATGTCGGGTAGATG	CGGAAAAGGCATTCGTCCAT	[44]
*hGDNF*	CACTGACTTGGGTCTGGGCTATGA	GTCTGCAACATGCCTGCCCTACTT	[45]
*hRET*	AGATTTCGGATTTCGGCTTGT	CCACAGCAGGACACCAAAAGA	[46]
*hRELA*	ATC CCA TCT TTG ACA ATCGTGC	CTG GTC CCG TGA AAT ACA CCT C	[42]
*hNF-KB1*	TGG ACA GCA AAT CCG CCC TG	TGT TGT AAT GAG TCG TCA TCC T	[47]
*hNFKBIA*	ATT CAC AGA GGA TGA GCT GCCC	TCCACATTCTTTTTGCCACTTTCC A	[41]
*hTNFA*	AGC CCA TGT TGT AGC AAA CC	GTT GGG CTG ATT GAT CTC AGC	[48]
*hTRAF6*	AGG GAC CCA GCT TTC TTT GT	GCC AAG TGA TTC CTC TGC AT	[49]
*hTGFB2*	CCA TCC CGC CCA CTT TCT AC	AGC TCA ATC CGT TGT TCA GGC	
*hTGFBR2*	CTA ACC TGC TGC CTG TGT GA	TCT GGA GCC ATG TAT CTT GC	[50]
*hERBB2*	AAT GCC AGG CAC TGT TTG	GTC CTT ATA GTG GGC ACA GG	[51]
*hERBB3*	AAG CTC TAC GAG AGG TGT GA	TGG GCA ATG GTA GAG TAG AG	[52]

## Data Availability

The data presented in this study are available on request from the corresponding author.

## Supplementary Materials

The following supporting information can be downloaded at: https://www.mdpi.com/article/10.3390/biom14080992/s1, Figure S1: An example of amplification plot of completed RT-qPCR reactions.
