# Supplementary figures and images for: Role of Inflammation and the NF-κB Signaling Pathway in Hirschsprung’s Disease
Source: Biomolecules. 2024 Aug 12;14(8):992. doi: 10.3390/biom14080992 (PMC11352745; doi:10.3390/biom14080992)

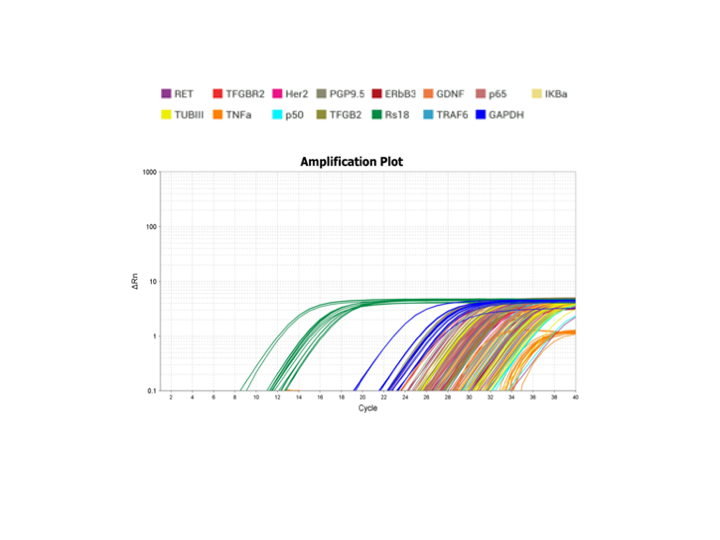

Supplement: Supplementary file 1 [file biomolecules-14-00992-s001.zip › biomolecules-3090640-supplementary.tiff]
